# Diabetic ketoacidosis in an adult with beta-ketothiolase deficiency (BKD) involving a novel *ACAT1* variant : first report of established diabetes in BKD and a review of the literature

**DOI:** 10.1186/s40842-024-00174-9

**Published:** 2024-06-10

**Authors:** Xi May Zhen, Stephen M. Twigg, Ted Wu, Eddy Tabet, Margaret J. McGill, Maria Constantino, Amali Mallawaarachchi, Connie Luo, Senthil Thillainadesan, Yusof Rahman, Jencia Wong

**Affiliations:** 1 Department of Endocrinology, Royal Prince Alfred Hospital, Sydney, NSW Australia; 2 Diabetes Centre, Royal Prince Alfred Hospital, Sydney, NSW Australia; 3 Sydney Medical School (Central), Faculty of Medicine and Health, University of Sydney, Sydney, NSW Australia; 4 Department of Endocrinology, Blacktown Hospital, Sydney, NSW Australia; 5 School of Medicine, Western Sydney University, Sydney, NSW Australia; 6 Clinical Genetics Service, Institute of Precision Medicine and Bioinformatics, Royal Prince Alfred Hospital, Sydney, NSW Australia; 7 Department of Genetic Medicine and ICPMR Chemical Pathology, Westmead Hospital, Sydney, NSW Australia

**Keywords:** Beta-ketothiolase deficiency, T2 deficiency, Diabetes, Ketoacidosis

## Abstract

**Background:**

Diabetes presenting in young adults is often challenging to classify. Diabetic ketoacidosis is typically seen in autoimmune type 1 diabetes mellitus and more rarely in young onset type 2 diabetes mellitus. Beta-ketothiolase deficiency (BKD) is a rare autosomal recessive condition affecting isoleucine catabolism and ketone body metabolism. BKD typically manifests in childhood as recurrent episodes of ketoacidosis, the frequency of which tends to reduce with age. There is a paucity of data with respect to the co-existence of persistent dysglycemia with BKD.

**Case presentation and literature review:**

We present a novel case of diabetes presenting as diabetic ketoacidosis in a 34-year-old man with BKD, with genetically confirmed compound heterozygosity for variants in *ACAT1*, including a novel *ACAT1* c.481T>C, p.(Tyr161His) variant. Diabetes in people with BKD presents unique diagnostic and management challenges. To further contextualize our findings, we conducted a comprehensive narrative review of the existing literature with respect to dysglycemia in those with BKD, especially in adulthood. There are no existing reports describing diabetes in adults with BKD. Stress hyperglycemia is not uncommon when children with BKD are acutely unwell, with several pediatric case reports describing short-lived hyperglycemia but normal HbA1c measurements during metabolic crises (indicating the absence of persistent hyperglycemia).

**Conclusions:**

This is the first report of diabetic ketoacidosis in an adult with BKD, with an elevated HbA1c consistent with persistent hyperglycemia. This case highlights the importance of checking HbA1c in people with BKD and hyperglycemia in order to uncover potential coexisting diabetes, facilitating timely management and preventing complications. Increased reporting on the longitudinal outcomes of those with rare metabolic disorders is essential for identifying potential associations with conditions like diabetes.

**Supplementary Information:**

The online version contains supplementary material available at 10.1186/s40842-024-00174-9.

## Background

**Fig. 1:**
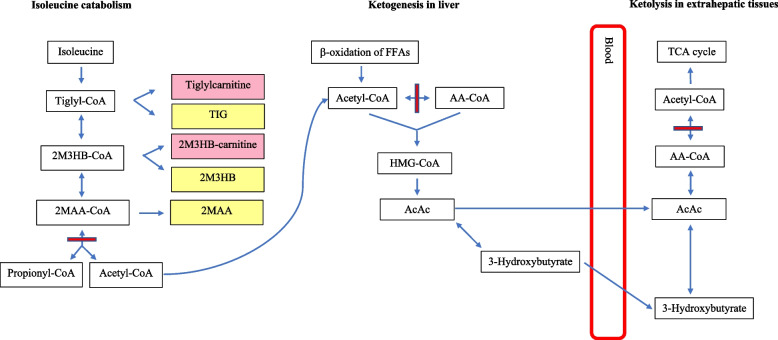
Summary of isoleucine catabolism and ketone metabolism. The short red bar denotes the site of action of beta-ketothiolase. 2M3HB = 2-methyl-3-hydroxybutyrate; 2M3HB- = 2-methyl-3-hydroxylbutyryl-; 2MAA = 2-methylacetoacetate; 2MAA- = 2‐methylacetoacetyl-; AA = acetoacetyl; AcAc = acetoacetate; CoA = Coenzyme A; FFA = free fatty acids; HMG = 3-hydroxy-3-methylglutaryl; TCA = tricarboxylic acid; TIG = tiglylglycine. Tiglylcarnitine and 2M3HB-carnitine (pink boxes) are characteristically elevated on blood acylcarnitine analysis in those with BKD. TIG, 2M3HB, and 2MAA (yellow boxes) are characteristically elevated on urinary organic acid analysis in those with BKD

Beta-ketothiolase deficiency (BKD) is a rare, autosomal recessive condition affecting isoleucine catabolism and ketone body metabolism (see Fig. 1) [1–4]. BKD is caused by mutations in the acetyl-CoA acetyltransferase 1 (*ACAT1*) gene [2]. Confirmatory testing for BKD can be performed by assessing for *ACAT1* gene mutations or conducting enzyme activity assays in patient cells [5]. Characteristic abnormalities on metabolic testing include elevated tiglylcarnitine and 2-methyl-3-hydroxybutyryl-carnitine on blood acylcarnitine analysis, and elevated tiglylglycine, 2-methyl-3-hydroxybutyrate, and 2-methylacetoacetate on assessment of urinary organic acid profile (see Fig. 1) [1, 6]. Although rare, it has been shown that even those with compound heterozygosity for null mutations (with no beta-ketothiolase activity) can have normal urinary organic acid, and normal blood and plasma acylcarnitine profiles when clinically well [7]. Those with disease-causing variants resulting in residual function may not be readily detected on urinary organic acid and blood acylcarnitine testing even during episodes of ketoacidosis [6].

Onset of symptoms is typically in early childhood, and BKD most commonly manifests as recurrent ketoacidosis episodes [2, 5]. Episodes of ketoacidosis are reported to become less frequent with age [2], and are rare after the age of 10 years [8]. Acute ketoacidosis episodes in people with BKD are usually triggered by infection, prolonged fasting, or consumption of a ketogenic diet [9]. Management of acute crises in those with BKD includes treatment with intravenous dextrose infusions to suppress ketogenesis (even if euglycemic), fluid and electrolyte replacement, and supportive measures as required [1]. Proposed strategies for the prevention of further ketoacidosis episodes include ensuring regular and frequent carbohydrate intake, and the avoidance of ketogenic diets [1]. Carnitine supplementation can be considered especially in those with carnitine deficiency [1].

While children with BKD are often normoglycemic when they present with ketoacidosis, here we report a case of diabetic ketoacidosis (DKA) in a 34 year old man with BKD. An elevated HbA1c at presentation in the diabetic range confirmed persistent hyperglycemia and genetic testing revealed compound heterozygosity for variants in *ACAT1*, including a novel *ACAT1* c.481T>C, p.(Tyr161His) variant. Additionally, we performed a comprehensive review of the existing literature with respect to dysglycemia in BKD, especially in adulthood, to further contextualize our findings.

## Case presentation

A 34-year-old Caucasian man presented to the Emergency Department with one week history of lethargy, thirst, and bilateral blurred vision. He was diagnosed in the United Kingdom with BKD at 18 months of age, and was managed with fasting avoidance and use of glucose polymer drinks when unwell. He had no further episodes of ketoacidosis since the index event. In adulthood, he was overweight (BMI 28.1 kg/m^2^) but otherwise well and did not take any regular medications. His parents (non-consanguineous) and siblings had no history of diabetes or other medical conditions, and they did not undergo testing for BKD following his diagnosis. His family history was significant for type 2 diabetes mellitus (T2DM) diagnosed in his paternal grandfather at an elderly age. Prior to this hospital admission, he had not fasted, had not consumed a ketogenic diet, and had no symptoms to suggest an intercurrent infection as a potential precipitant.

In the Emergency Department, initial blood tests were consistent with mixed DKA/hyperglycemic hyperosmolar state (HHS) and hypertriglyceridemia (see Table 1). There was no evidence of an infective or other acute trigger for the DKA/HHS, and the ECG demonstrated sinus tachycardia without features of ischemia. The diabetic ketoacidosis and hypertriglyceridemia normalised with an insulin infusion and supportive rehydration. He recovered well and was discharged from the hospital on a basal-bolus insulin regimen. On serial review in our outpatient diabetes clinic, he continued to require a total daily insulin dose of ~0.4 units/kg of body weight at > 12 months post-admission.

**Table 1:** Initial blood test results at presentation to the Emergency Department

	**Result**	**Reference Range**
Plasma glucose (non-fasting)	46 mmol/L	4.0 to 7.7 mmol/L
Capillary ketones	6.2 mmol/L	< 0.6 mmol/L
Plasma C-peptide (non-fasting)	165 pmol/L	200 to 1200 pmol/L (fasting range)
HbA1c	11.5% (102 mmol/mol)	3.5 to 6.0 % (15 to 42 mmol/mol)
Serum sodium	130 mmol/L	135 to 145 mmol/L
Serum osmolality	325 mmol/kg	280 to 300 mmol/kg
Anion Gap [Na+] - [Cl-] - [HCO3-]	21 mmol/L	4 to 13 mmol/L
Venous blood pH	7.29	7.3 to 7.4
Venous blood bicarbonate	13 mmol/L	22 to 32 mmol/L
Venous base excess	Minus 12 mmol/L	Minus 3.0 to 3.0 mmol/L
Venous blood lactate	1 mmol/L	0 to 2.0 mmol/L
Serum triglyceride level	17.9 mmol/L	≤ 2.5 mmol/L
Serum total cholesterol level	7.1 mmol/L	≤ 5.2 mmol/L
Serum lipase	40 u/L	13 to 60 u/L

Antibodies against glutamic acid decarboxylase, islet tyrosine phosphatase 2, insulin, and zinc transporter 8 were negative. At presentation, the C peptide level was low at 165 pmol/L when his blood glucose level was severely elevated at 46 mmol/L. A monogenic diabetes gene panel (Blueprint Genetics MODY Flex Panel version 1, 09 January 2020) was negative. Abdominal ultrasound did not reveal any pancreas or liver abnormalities. There was no evidence of diabetic nephropathy or retinopathy. His blood pressure and serum lipid profile were persistently normal following discharge from hospital. The urine metabolic screen (including targeted metabolomics profile by liquid chromatography with tandem mass spectrometry, organic acids by gas chromatography mass spectrometry**,** and a mucopolysaccharides screen by spectroscopy) was normal, though this was performed when he was clinically well following his acute presentation.

**Fig. 2:**
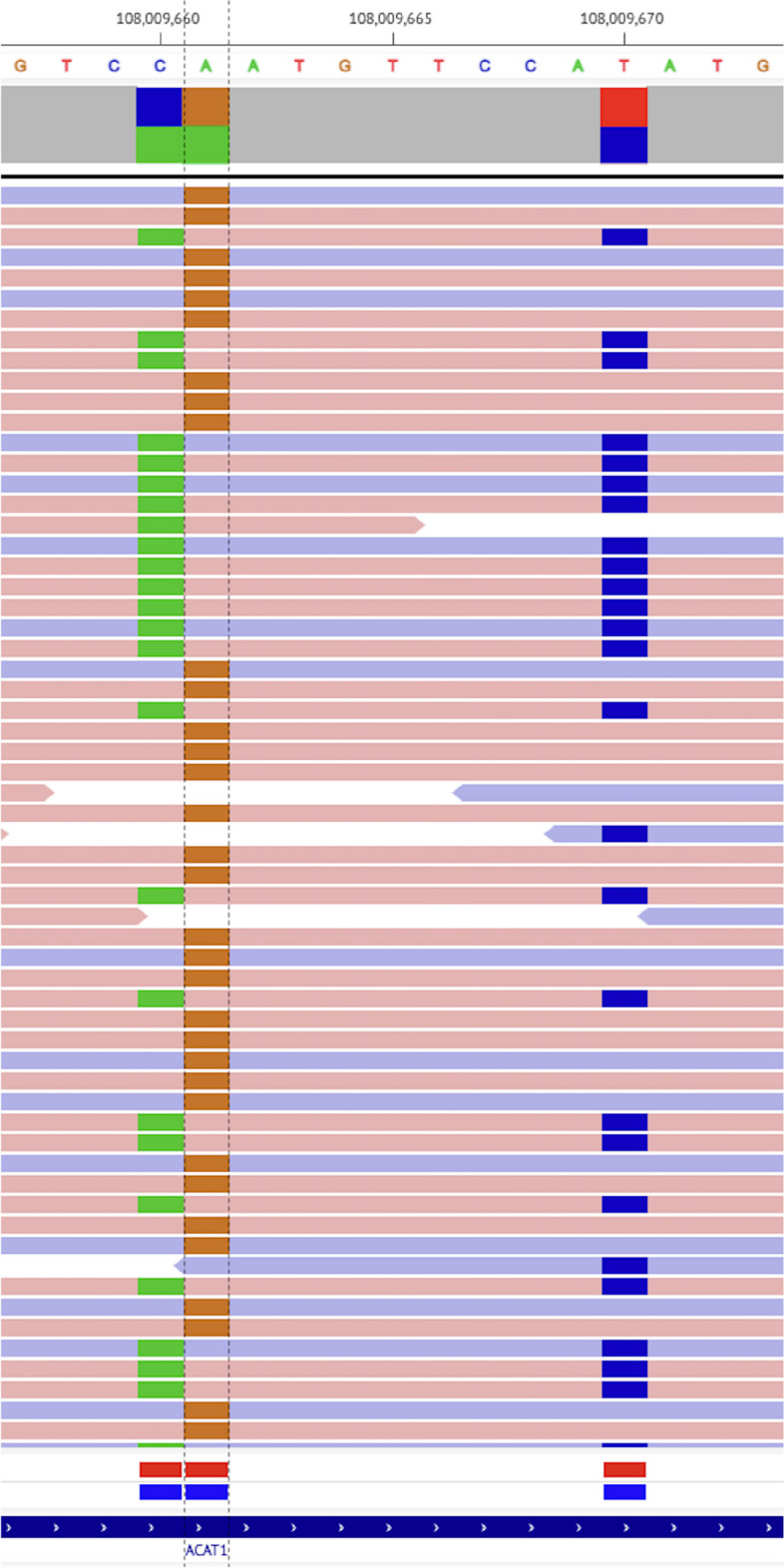
Two detected *ACAT1* variants (next-generation sequencing data visualisation with Integrative Genomics Viewer). Left (brown): Chr11(GrCh37):108009661 NM_000019.3 c.472A>G, p.(Asn158Asp). Right (blue): Chr11(GrCh37)11:108009670 NM_000019.3 c.481T>C, p.(Tyr161His)

Genetic testing was performed to confirm the diagnosis of BKD (as the initial diagnosis was made overseas > 30 years ago and could not be confirmed via his medical records). He was confirmed to be a compound heterozygote for two missense variants in *ACAT1* (see Fig. 2). He was heterozygous for the *ACAT1* c.472A>G, p.(Asn158Asp) variant which affects a highly conserved amino acid. All *in silico* analyses used predict this variant to be damaging to protein structure and function. This variant was previously reported in combination with other disease-causing *ACAT1* variants in at least 3 individuals with BKD [10–12]. Previous *in vivo* expression analysis of Asn158Asp demonstrated no residual enzyme activity [10]. The present patient was also heterozygous for the novel *ACAT1* c.481T>C, p.(Tyr161His) variant. This variant has not been described in the medical literature or reported in disease-related variation databases such as ClinVar [13] or the Human Gene Mutation Database [14]. This variant affects a highly conserved amino acid. *In silico* analyses utilised predict this variant to be damaging to protein structure and function, and this variant was classified as likely pathogenic.

## Case discussion

This is the first report of ketoacidosis and severe and persistent hyperglycemia, with an elevated HbA1c consistent with diabetes in an adult with BKD. The present patient was a compound heterozygote for the novel *ACAT1* c.481T>C, p.(Tyr161His) variant. It is unknown whether this variant may play a role in the development of diabetes.

The etiology of this patient's diabetes is a key question. The specific diabetes phenotype is often challenging to ascertain in youth and young adults, especially in the context of a high background prevalence of obesity in this population [15–17]. While BKD may have predisposed him to the development of ketoacidosis, there were no identifiable triggers such as consuming a ketogenic diet or an intercurrent illness. There was clear evidence of relative insulin deficiency at presentation where the C peptide level was low in the context of a severely elevated serum blood glucose level of 46 mmol/L. Glucotoxicity likely exacerbated beta cell dysfunction at presentation, and his endogenous insulin secretory capacity may improve with optimisation of his glycemia. MODY was excluded by genetic testing and the differential diagnosis for this diabetes case is discussed below.

Ketosis-prone young adult-onset T2DM is a possibility. However, young onset T2DM in people of Caucasian background is usually strongly associated with obesity and metabolic syndrome [16, 18, 19]. This patient's BMI was in the overweight but not the obese category, and he lacked the usual comorbid risk factors associated with young onset T2DM. He did not have any first-degree relatives with diabetes. He did not have any physical features suggestive of insulin resistance, and his total daily insulin requirement remains relatively low at ~0.4 units/kg of body weight. Young people with T2DM can sometimes present with ketosis and mimic “antibody-negative” type 1 diabetes mellitus (T1DM). Ketosis-prone T2DM has been documented in all non-Caucasian populations in which it has been sought [20]. The World Health Organisation (WHO) Classification of Diabetes Mellitus (2019) recommends considering the possibility of ketosis-prone T2DM in adults of all ethnicities (except Caucasian populations) who present with ketosis, but otherwise have most features of T2DM. Given our patient is Caucasian, ketosis-prone T2DM is less likely.

Latent autoimmune diabetes in adults (LADA) or T1DM is also a possibility, though less likely given this patient is Caucasian and there was no evidence of islet cell autoantibodies. Previous studies have suggested that approximately 5 to 15% of individuals with a clinical phenotype consistent with T1DM are autoantibody negative [21, 22]. An observational study conducted in 3312 individuals with clinical T1DM from the United Kingdom reported that autoantibody positivity was less common with increasing age, in males versus females, and in non-white versus white ethnicity [23]. This study also reported that autoantibody-negative individuals had higher BMI when compared with autoantibody-positive individuals [23], though a previous study in children with clinical T1DM did not report an association between autoantibody status and BMI [24]. There is a lack of consensus regarding what was traditionally referred to as “non-immune mediated T1DM"; it is notable that autoantibody-negative T1DM has been removed from the WHO (2019) classification of diabetes as this terminology was "not frequently used nor is it clinically helpful" [25]. This patient’s presentation and biochemical profile was also not consistent with fulminant T1DM which is characterised by virtually no C-peptide secretion, elevated pancreatic enzyme levels, and usually close to normal HbA1c [26, 27]. There were no features identified on clinical history, biochemical testing, and imaging of the pancreas to suggest pancreatogenic diabetes.

Organic acids can accumulate in tissues over time, and it has been postulated that the accumulation of organic acids in the pancreas may predispose to diabetes by impairing the structure and normal functionality of the pancreas [28]. Thus, a specific BKD-related diabetes remains a possibility in our patient*.* Regardless of the precise etiology, the diagnosis and management of intercurrent diabetes is challenging in those with rare metabolic conditions like BKD. BKD predisposes this patient to ketoacidosis and the trajectory of his beta cell function is unclear. Therefore, regardless of the specific etiology of his diabetes, continuation on a basal-bolus insulin regimen at this stage (with doses dictated by his ongoing glycemia), would be important to help prevent further episodes of ketoacidosis*.* We also recommend avoiding the use of sodium-glucose co-transporter 2 (SGLT-2) inhibitors in the context of BKD, given the risk of ketoacidosis. From a BKD management perspective in the context of his diabetes, prevention of ketoacidosis is reliant on strategies to ensure regular and frequent carbohydrate intake matched by adequate insulin administration, as well as the avoidance of ketogenic diets and ensuring that an appropriate sick day plan is in place.

## Literature review: dysglycemia, diabetes and BKD

We searched Medline via OvidSP (inception to 10 Jan 2024) for articles published in the English language on BKD in humans. The following search terms were used: "beta-ketothiolase deficiency" or "β-ketothiolase deficiency" or "T2 deficiency" or "3-ketothiolase deficiency" or "3-oxothiolase deficiency" or "alpha methylacetoacetic aciduria" or "alpha-methyl-acetoacetyl-Coenzyme A thiolase deficiency" or "alpha-methyl-acetoacetyl-CoA thiolase deficiency" or "2-methylacetoacetyl-coenzyme A thiolase deficiency" or "2-methylacetoacetyl-CoA thiolase deficiency" or "mitochondrial methylacetoacetyl-coenzyme A thiolase deficiency" or "mitochondrial methylacetoacetyl-CoA thiolase deficiency" or "mitochondrial acetoacetyl-Coenzyme A thiolase deficiency" or "mitochondrial acetoacetyl-CoA thiolase deficiency". The reference lists of relevant articles were also screened.

We identified 104 articles as shown in Appendix 1 Tables A to C. There were 7 articles [5, 6, 29–33] that included 11 individual adults with BKD (see Appendix 1, Table A). Out of these 11 adults, 3 cases [30, 31, 33] were pregnant women with BKD, 3 cases [29] were only briefly mentioned in a paper focused on molecular studies, 4 cases [6] only had their adult ages mentioned in a review article, and 1 case [32] was a father who was found to have BKD with screening performed following his son's diagnosis with BKD. The review of the literature confirms that there are no published papers to date (in the English language) describing diabetes or DKA in adults with BKD. In fact, the papers that included adults with BKD did not report blood glucose level (BGL) measurements in adulthood. At present, there are 14 original and review papers describing hyperglycemia ≥ 11.1 mmol/L in children with BKD (listed in Appendix 1, Table B). While some of these pediatric cases with hyperglycemia mimicked DKA, none had longstanding hyperglycemia and an established diagnosis of diabetes. Al-Hakami et al. described a 1-year-old boy with BKD who presented with a significantly elevated BGL of 22 mmol/L and ketoacidosis, mimicking T1DM [34]. This child had a normal HbA1c of 4.4% at presentation and did not have autoimmune diabetes [34]. Insulin therapy was only required for a few months before gradual recovery of pancreatic function, and the child was subsequently able to remain off exogenous insulin with maintenance of normal BGLs when well [34]. Patra et al. also described an 8-month-old boy who presented with shock and severe respiratory distress, and was noted to have severe metabolic acidosis and intermittent hyperglycemia and hypoglycemia [35]. The presentation, however, was not consistent with established diabetes given the normal HbA1c of 5.4% (3.5–6.0%) and the infant only required insulin initially [35]. Fontaine et al. described a 4-year-old boy who was noted to have a BGL of 12.7 mmol/L and severe metabolic acidosis with ketosis when he presented with an acute illness [36]. The diagnosis was initially geared towards DKA but this was quickly rejected and he was subsequently diagnosed with BKD [36]. Nakagawa et al. described a 13-month-old boy with BKD who presented with a diarrhoeal illness and was noted to have severe hyperglycemia (BGL 38.6 mmol/L), though the hyperglycemia was only transient [37]. In general, transient hyperglycaemia is generally <15mmol/L during acute episodes when present [5, 38]. Although hyperglycemia in association with ketoacidosis has been described, there are no reports of persistent hyperglycemia requiring ongoing treatment in children. Nor are there reports of the natural history of BKD into adulthood with respect to recurrence of dysglycemia.

## Conclusions

We need to keep in mind that individuals with rare metabolic conditions are not protected from common metabolic conditions (like diabetes), particularly as improved management has resulted in many of these individuals surviving into adulthood. We have provided the first report of diabetes in an adult with BKD. This case highlights the importance of measuring HbA1c in those with BKD and hyperglycemia to assess for potential coexisting diabetes, and to allow timely management and prevention of complications. Conversely, in the context of many cases of BKD likely being missed on newborn screening [7], and previous reports of adults with BKD who were asymptomatic and only diagnosed with family screening [5, 32], our case suggests that co-existent BKD could be considered in the evaluation of ketosis-prone T2DM in youth and young adults.

There is a need to collect longitudinal data into adulthood for those with rare metabolic conditions such as BKD, so that a rigorous evidence-base can be developed to further inform management recommendations and also to allow the identification of any potential association with other conditions such as diabetes.

### Supplementary Information

**Supplementary file 1:** **Appendix 1.** Reporting of dysglycemia by articles describing people with BKD. Table A. Reporting of dysglycemia by articles including at least 1 adult (≥ 18 years) with BKD. Table B. Reporting of dysglycemia by articles describing children with BKD (or if age not available) where hyperglycemia ≥ 11.1 mmol/L was described. Table C. Reporting of dysglycemia by articles describing children with BKD (or if age not available) where there was no description of hyperglycemia ≥ 11.1 mmol/L.

## Data Availability

Data sharing is not applicable to this article as no new datasets were generated or analysed during the current study.

## Abbreviations

ACAT1: Acetyl-CoA acetyltransferase 1
BKD: Beta-ketothiolase deficiency
DKA: Diabetic ketoacidosis
LADA: Latent autoimmune diabetes in adults
MODY: Maturity-onset diabetes of the young
T2DM: Type 2 diabetes mellitus
T1DM: Type 1 diabetes mellitus
WHO: World Health Organization
