# Supplementary material for: Diabetic ketoacidosis in an adult with beta-ketothiolase deficiency (BKD) involving a novel ACAT1 variant : first report of established diabetes in BKD and a review of the literature
Source: Clin Diabetes Endocrinol. 2024 Jun 10;10:17. doi: 10.1186/s40842-024-00174-9 (PMC11163784; doi:10.1186/s40842-024-00174-9)
Supplement: Supplementary file 1 — Appendix 1. Reporting of dysglycemia by articles describing people with BKD. Table A. Reporting of dysglycemia by articles including at least 1 adult (≥ 18 years) with BKD. Table B. Reporting of dysglycemia by articles describing children with BKD (or if age not available) where hyperglycemia ≥ 11.1 mmol/L was described. Table C. Reporting of dysglycemia by articles describing children with BKD (or if age not available) where there was no description of hyperglycemia ≥ 11.1 mmol/L. [file 40842_2024_174_MOESM1_ESM.docx]

**Appendix 1. Reporting of dysglycemia by articles describing people with BKD**

**Table A. Reporting of dysglycemia by articles including at least 1 adult (≥ 18 years) with BKD**

| **Article*** | **Article type** | **No. of patients** | **Adult included** | **BMI**  **(kg/m^2^)** | **Weight** | **Peak glucose mmol/L** | **Lowest glucose mmol/L** | **Oldest age reported** | **Diabetes reported** |
| --- | --- | --- | --- | --- | --- | --- | --- | --- | --- |
| Fukao T, Yamaguchi S, Scriver CR, Dunbar G, Wakazono A, Kano M, et al. Molecular studies of mitochondrial acetoacetyl-coenzyme A thiolase deficiency in the two original families. Hum Mutat. 1993;2:214–20. | Original case and further analyses on previously published cases | 3 | Yes  3 adults | N/A | N/A | N/A | N/A | The article briefly mentioned that the 3 patients are now > 18 years (unclear exact ages). The 3 patients "have developed normally" and "are now gainfully employed adults". | No |
| Grünert SC, Sass JO. 2-methylacetoacetyl-coenzyme A thiolase (beta-ketothiolase) deficiency: one disease - two pathways. Orphanet J Rare Dis. 2020;15(1):106 | Review | 244 | Yes  1 adult | N/A | N/A | N/A | N/A | 36 years  from case reported by Schutgens et al. (1982) also included in this table. | No |
| Hori T, Yamaguchi S, Shinkaku H, Horikawa R, Shigematsu Y, Takayanagi M, et al. Inborn errors of ketone body utilization. Pediatrics International. 2015;57(1):41-8. | Review | 8 | Yes  4 adults | N/A | N/A | N/A | N/A | 33 years.  No information on wellbeing, article just included a table that listed the patient's current age in 2014. | No |
| Kayani R, Botros S, Moore P. Beta-ketothiolase deficiency and pregnancy. Int J Obstet Anesth. 2013;22:260–1. | Original case | 1 | Yes  1 adult | N/A | N/A | N/A | N/A | 32 years | No |
| Kiran S, Murshedi FA, Jabri SA, Devi MN. Alpha-Methylacetoacetic Aciduria in an Rh-Negative Pregnant Omani Woman With Breech Presentation Delivered With Favourable Outcome. J Obstet Gynaecol Can. 2019;41(4):492-4. | Original case | 1 | Yes  1 adult | N/A | N/A | N/A | N/A | 22 years | No |
| Schutgens RB, Middleton B, vd Blij JF, Oorthuys JW, Veder HA, Vulsma T, et al. Beta-ketothiolase deficiency in a family confirmed by in vitro enzymatic assays in fibroblasts. Eur J Pediatr. 1982;139:39–42. | Original case | 2 | Yes  1 adult | N/A | 36 year old father was "healthy". | N/A | N/A | 36 years. | No |
| Sewell AC, Herwig J, Wiegratz I, Lehnert W, Niederhoff H, Song XQ, et al. Mitochondrial acetoacetyl-CoA thiolase (beta-ketothiolase) deficiency and pregnancy. J Inherit Metab Dis. 1998;21:441–2. | Original case | 1 | Yes  1 adult | 35.5 | 87.5 | N/A | N/A | 25 years | No |

*Please note articles were sorted alphabetically by the first author's surname, then by year of publication if the first authors' surnames were identical, then alphabetically by the second author's surname.

**Table B. Reporting of dysglycemia by articles describing children with BKD (or if age not available) where hyperglycemia ≥ 11.1 mmol/L was described**

| **Article**  **(sorted alphabetically)** | **Article type** | **No. of patients** | **Adult included** | **BMI**  **(kg/m^2^)** | **Weight** | **Peak glucose mmol/L** | **Lowest glucose mmol/L** | **Oldest age reported** | **Diabetes reported** |
| --- | --- | --- | --- | --- | --- | --- | --- | --- | --- |
| Abdelkreem E, Otsuka H, Sasai H, Aoyama Y, Hori T, Abd El Aal M, et al. Beta-Ketothiolase Deficiency: Resolving Challenges in Diagnosis. Journal of Inborn Errors of Metabolism and Screening. 2016;4:2326409816636644. | Review of topic | No case discussion | N/A | N/A | N/A | 14.1 mmol/L  from case reported by Riudor et al. (1995) also included in this table. | N/A | N/A | N/A |
| Abdelkreem E, Akella RRD, Dave U, Sane S, Otsuka H, Sasai H, et al. Clinical and Mutational Characterizations of Ten Indian Patients with Beta-Ketothiolase Deficiency. JIMD Rep. 2017;35:59–65. | Original case | 10 | No | N/A | N/A | 14.7 | 1.4 | "5.5 years" | No |
| Al-Hakami A, Alghamdi M, Sumaily K, Al-Khalifah R. A challenging diagnosis of B-ketothiolase deficiency mimicking type 1 diabetes mellitus. Journal of Nature and Science of Medicine. 2021;4(1):74-7. | Original case | 1 | No | N/A | -2.3 SD | 22.0 | 2.2 | 2 years | No. Initially diagnosed with diabetic ketoacidosis but was subsequently weaned off insulin with maintenance of normal BGLs when well. |
| Arica V, Arica SG, Dag H, Onur H, Obut O, Gülbayzar S. Beta-ketothiolase deficiency brought with lethargy: case report. Hum Exp Toxicol. 2011;30:1724–7. | Original case | 1 | No | N/A | Weight "between 25th and 50th centile" | 14.6 | N/A | 9 months | No |
| Fontaine M, Briand G, Ser N, Armelin I, Rolland MO, Degand P, et al. Metabolic studies in twin brothers with 2-methylacetoacetyl-CoA thiolase deficiency. Clin Chim Acta. 1996;255:67–83. | Original case | 2 | No | N/A | "staturo-ponderal delay".  Weight 2 to 2.5 SD. Height 0.8 to 1.5 SD | 12.7 | N/A | 7 years | No. One patient initially thought to have DKA but diagnosis quickly rejected |
| Fukao, T . Beta-ketothiolase deficiency. Orphanet encyclopedia, September 2001. Updated September 2004. Web site. http://www.orpha.net/data/patho/GB/uk-T2.pdf. Accessed Jan 10, 2024. | Orphanet Encyclopedia | N/A | N/A | N/A | N/A | 12.7  from case by Fontaine et al. (1996) which is also included in this table. The study by Riudor et al. (1995) was also cited though the higher BGL of 14.1 mmol/L from that study was not included. | 0.6 | N/A | No |
| Fukao T, Nakamura H, Nakamura K, Perez-Cerda C, Baldellou A, Barrionuevo CR, et al. Characterization of six mutations in five Spanish patients with mitochondrial acetoacetyl-CoA thiolase deficiency: effects of amino acid substitutions on tertiary structure. Mol Genet Metab. 2002;75:235–43. | Original case and previously published cases | 5 | No | N/A | N/A | 17.4 | 1.6 | 13 years | No |
| Fukao T, Nguyen HT, Nguyen NT, Vu DC, Can NTB, Pham ATV, et al. A common mutation, R208X, identified in Vietnamese patients with mitochondrial acetoacetyl-CoA thiolase (T2) deficiency. Mol Genet Metab. 2010;100:37–41. | Original case | 10 | No | N/A | N/A | 19.4 | 2.0 | 14 years | No |
| Fukao T, Sasai H, Aoyama Y, Otsuka H, Ago Y, Matsumoto H, et al. Recent advances in understanding beta-ketothiolase (mitochondrial acetoacetyl-CoA thiolase, T2) deficiency. Journal of Human Genetics. 2019;64(2):99-111. | Review | 135 | No | N/A | N/A | 23.3  from case by Nguyen et al. (2017) also included in this table. | 1.6 | 17 years | No |
| Köse, Melis Demir, Canda, Ebru, Kagnici, Mehtap, İşgüder, Rana, Ünalp, Aycan, Uçar, Sema Kalkan, et al. Two Siblings with Beta-Ketothiolase Deficiency: One Genetic Defect Two Different Pictures. J Pediatr Res. 2016;3:113–6. | Original case | 2 | No | N/A | N/A | 12.2 | N/A | 7 years | No |
| Nakagawa T, Kuroda K, Fujiwara I, Ohura T. Emergent treatment using glucose/insulin infusing for ketoacidosis in T2 deficiency. Pediatrics International. 2021;63(4):482-4. | Original case | 1 | No | N/A | N/A | 38.6 | 4.9 | 13 months | No. "Although hyperglycemia with extremely elevated BG levels were seen during the treatment, hyperglycemia was transient" |
| Nguyen KN, Abdelkreem E, Colombo R, Hasegawa Y, Can NTB, Bui TP, et al. Characterization and outcome of 41 patients with beta-ketothiolase deficiency: 10 years’ experience of a medical center in northern Vietnam. J Inherit Metab Dis. 2017;40:395–401. | 10 year retrospective data with original cases | 41 | No | N/A | "-2.5 SD to 1.5 SD" | 23.3 | 2.0 | 11 years | No |
| Patra B, Rangrajan S, Kotekar S, Malhotra V. A rare case of β-ketothiolase deficiency presenting as mimicker of diabetic ketoacidosis. J Pediatr Endocrinol Diabetes 2023;3:78-81. | Original case | 1 | No | N/A | "-1 SD and −2 SD" | 26.2 | 1.6 | 8 months | No. HbA1C was normal at 5.4% (3.5–6.0%) |
| Riudor E, Ribes A, Perez-Cerda C, Arranz JA, Mora J, Yeste D, et al. Metabolic coma with ketoacidosis and hyperglycaemia in 2-methylacetoacetyl-CoA thiolase deficiency. J Inherit Metab Dis. 1995;18:748–9. | Original case | 1 | No | N/A | N/A | 14.1 | N/A | 14 months | No |

*Please note articles were sorted alphabetically by the first author's surname, then by year of publication if the first authors' surnames were identical, then alphabetically by the second author's surname.

**Table C. Reporting of dysglycemia by articles describing children with BKD (or if age not available) where there was no description of hyperglycemia ≥ 11.1 mmol/L**

| **Article*** | **Article type** | **No. of patients** | **Adult included** | **BMI**  **(kg/m^2^)** | **Weight** | **Peak glucose mmol/L** | **Lowest glucose mmol/L** | **Oldest age reported** | **Diabetes reported** |
| --- | --- | --- | --- | --- | --- | --- | --- | --- | --- |
| Abdelkreem E, Alobaidy H, Aoyama Y, Mahmoud S, Abd El Aal M, Fukao T. Two Libyan siblings with beta-ketothiolase deficiency: A case report and review of literature. Egyptian Journal of Medical Human Genetics. 2017;18(2):199-203. | Original case | 2 | No | N/A | Patient 1 died at 14 months.  Patient 2: " age-appropriate growth and development" | N/A | 0.95 | 7.5 years | No |
| Abdelkreem E, Harijan RK, Yamaguchi S, Wierenga RK, Fukao T. Mutation update on ACAT1 variants associated with mitochondrial acetoacetyl-CoA thiolase (T2) deficiency. Human Mutation. 2019;40(10):1641-63. | Review | 149 | N/A | N/A | N/A | N/A | N/A | N/A | No |
| Akella RRD, Aoyama Y, Mori C, Lingappa L, Cariappa R, Fukao T. Metabolic encephalopathy in beta-ketothiolase deficiency: the first report from India. Brain Dev. 2014;36:537–40. | Original case | 1 | No | N/A | 85th centile | N/A | 1.8 | 2 years 6 months | No |
| Alfadhel M, Babiker A. Inborn errors of metabolism associated with hyperglycaemic ketoacidosis and diabetes mellitus: narrative review. Sudan J Paediatr. 2018;18(1):10-23. | Review | N/A | No | N/A | N/A | N/A | N/A | N/A | No |
| Alijanpour M, Sasai H, Abdelkreem E, Ago Y, Soleimani S, Moslemi L, et al. Beta-ketothiolase deficiency: A case with unusual presentation of nonketotic hypoglycemic episodes due to coexistent probable secondary carnitine deficiency. JIMD Rep. 2019;46:23–7. | Original case | 1 | No | N/A | -3.5 SD | 6.3 | 1.3 | 5 years 5 months | No |
| Aramaki S, Lehotay D, Sweetman L, Nyhan WL, Winter SC, Middleton B. Urinary excretion of 2-methylacetoacetate, 2-methyl-3-hydroxybutyrate and tiglylglycine after isoleucine loading in the diagnosis of 2-methylacetoacetyl-CoA thiolase deficiency. J Inherit Metab Dis. 1991;14:63–74. | Original case | 5 | No | N/A | ranging from "failure to thrive" to 50th centile | N/A | N/A | 19 months | No |
| Bancel LP, Germain N, Guemann AS, Joncquel Chevalier Curt M, Dessein AF. Abnormal Ketone Bodies in a 22-Month-Old Boy Presenting with Recurrent Vomiting and Metabolic Acidosis. Clin Chem. 2019;65(11):1460-2. | Original case | 1 | No | N/A | N/A | N/A | 2.3 mmol/L | 22 months | No |
| Bennett MJ, Littlewood JM, MacDonald A, Pollitt RJ, Thompson J. A case of beta-ketothiolase deficiency. J Inherit Metab Dis. 1983;6:157. | Original case | 1 | No | N/A | 10kg | N/A | N/A | 17 months | No |
| Buhaş D, Bernard G, Fukao T, Décarie J-C, Chouinard S, Mitchell GA. A treatable new cause of chorea: beta-ketothiolase deficiency. Mov Disord. 2013;28:1054–6. | Original case | 1 | No | N/A | N/A | N/A | N/A | 17 years | No |
| Catanzano F, Ombrone D, Di Stefano C, Rossi A, Nosari N, Scolamiero E, et al. The first case of mitochondrial acetoacetyl-CoA thiolase deficiency identified by expanded newborn metabolic screening in Italy: the importance of an integrated diagnostic approach. J Inherit Metab Dis. 2010;33 Suppl 3:S91-94. | Original case | 2 | No | N/A | birth weight 25th to 50th centile | N/A | N/A | 18 months | No |
| Cromby CH, Manning NJ, Pollitt RJ, Powell S, Bennett MJ. 6-Methyluracil excretion in 2-methylacetoacetyl-CoA thiolase deficiency and in two children with an unexplained recurrent ketoacidaemia. J Inherit Metab Dis. 1994;17:81–4. | Original case | 4 | No | N/A | N/A | N/A | N/A | 4 years | No |
| Daum RS, Lamm PH, Mamer OA, Scriver CR. A “new” disorder of isoleucine catabolism. Lancet. 1971;2:1289–90. | Original case | 1 | No | N/A | N/A | N/A | N/A | 6 years | No |
| Daum RS, Scriver CR, Mamer OA, Delvin E, Lamm P, Goldman H. An inherited disorder of isoleucine catabolism causing accumulation of alpha-methylacetoacetate and alpha-methyl-beta -hydroxybutyrate, and intermittent metabolic acidosis. Pediatr Res. 1973;7:149–60. | Original case | 2 pedigrees  (3 presumed homozygotes) | No | N/A | "thin child" | N/A | N/A | 8 years | No |
| Estrella J, Wilcken B, Carpenter K, Bhattacharya K, Tchan M, Wiley V. Expanded newborn screening in New South Wales: missed cases. J Inherit Metab Dis. 2014;37:881–7. | Original case | 15 | No | N/A | N/A | N/A | N/A | N/A | No |
| Frazier DM, Millington DS, McCandless SE, Koeberl DD, Weavil SD, Chaing SH, et al. The tandem mass spectrometry newborn screening experience in North Carolina: 1997-2005. J Inherit Metab Dis. 2006;29:76–85. | Original case | 2 | No | N/A | N/A | N/A | N/A | N/A | No |
| Fukao T, Yamaguchi S, Wakazono A, Orii T, Hoganson G, Hashimoto T. Identification of a novel exonic mutation at -13 from 5’ splice site causing exon skipping in a girl with mitochondrial acetoacetyl-coenzyme A thiolase deficiency. J Clin Invest. 1994;93:1035–41. | Original case | 1 | No | N/A | N/A | N/A | N/A | 3 years | No |
| Fukao T, Song XQ, Yamaguchi S, Orii T, Wanders RJ, Poll-The BT, et al. Mitochondrial acetoacetyl-coenzyme A thiolase gene: a novel 68-bp deletion involving 3’ splice site of intron 7, causing exon 8 skipping in a Caucasian patient with beta-ketothiolase deficiency. Hum Mutat. 1995;5:94–6. | Original case | 1 | No | N/A | N/A | N/A | N/A | 4 years | No |
| Fukao T, Yamaguchi S, Orii T, Hashimoto T. Molecular basis of beta-ketothiolase deficiency: mutations and polymorphisms in the human mitochondrial acetoacetyl-coenzyme A thiolase gene. Hum Mutat. 1995;5:113–20. | Case series including published and unpublished cases | 17 | No | N/A | N/A | N/A | N/A | 48 months | No |
| Fukao T, Kodama A, Aoyanagi N, Tsukino R, Uemura S, Song XQ, et al. Mild form of beta-ketothiolase deficiency (mitochondrial acetoacetyl-CoA thiolase deficiency) in two Japanese siblings: identification of detectable residual activity and cross-reactive material in EB-transformed lymphocytes. Clin Genet. 1996;50:263–6. | Original case | 2 | No | N/A | N/A | N/A | N/A | 6 years 9 months | No |
| Fukao T, Song XQ, Yamaguchi S, Kondo N, Orii T, Matthieu JM, et al. Identification of three novel frameshift mutations (83delAT, 754insCT, and 435 + 1G to A) of mitochondrial acetoacetyl-coenzyme A thiolase gene in two Swiss patients with CRM-negative beta-ketothiolase deficiency. Hum Mutat. 1997;9:277–9. | Original case | 2 | No | N/A | N/A | N/A | N/A | 8.5 years | No |
| Fukao T, Scriver CR, Kondo N, t2 Collaborative Working Group. The clinical phenotype and outcome of mitochondrial acetoacetyl-CoA thiolase deficiency (beta-ketothiolase or T2 deficiency) in 26 enzymatically proved and mutation-defined patients. Mol Genet Metab. 2001;72:109–14. | Original case | 26 | No | N/A | N/A | N/A | < 2.5 | 16 years | No |
| Fukao T, Matsuo N, Zhang GX, Urasawa R, Kubo T, Kohno Y, et al. Single base substitutions at the initiator codon in the mitochondrial acetoacetyl-CoA thiolase (ACAT1/T2) gene result in production of varying amounts of wild-type T2 polypeptide. Hum Mutat. 2003;21:587–92. | Original case | 1 | No | N/A | N/A | N/A | N/A | N/A | No |
| Fukao T, Zhang GX, Sakura N, Kubo T, Yamaga H, Hazama A, et al. The mitochondrial acetoacetyl-CoA thiolase (T2) deficiency in Japanese patients: urinary organic acid and blood acylcarnitine profiles under stable conditions have subtle abnormalities in T2-deficient patients with some residual T2 activity. J Inherit Metab Dis. 2003;26:423–31. | Original case | 5 | No | N/A | N/A | N/A | N/A | 23 months | No |
| Fukao T, Zhang G, Rolland M-O, Zabot M-T, Guffon N, Aoki Y, et al. Identification of an Alu-mediated tandem duplication of exons 8 and 9 in a patient with mitochondrial acetoacetyl-CoA thiolase (T2) deficiency. Mol Genet Metab. 2007;92:375–8. | Original case | 1 | No | 22.4 (adult) | 56 kg  (adult) | N/A | 3.1 | 21 years 6 months | No |
| Fukao T, Boneh A, Aoki Y, Kondo N. A novel single-base substitution (c.1124A>G) that activates a 5-base upstream cryptic splice donor site within exon 11 in the human mitochondrial acetoacetyl-CoA thiolase gene. Mol Genet Metab. 2008;94:417–21. | Original case | 1 | No | N/A | N/A | N/A | 2.3 | 2 years 9 months | No |
| Fukao T, Horikawa R, Naiki Y, Tanaka T, Takayanagi M, Yamaguchi S, et al. A novel mutation (c.951C>T) in an exonic splicing enhancer results in exon 10 skipping in the human mitochondrial acetoacetyl-CoA thiolase gene. Mol Genet Metab. 2010;100:339–44. | Original case | 1 | No | N/A | N/A | N/A | 0.5 | 7 months | No |
| Fukao T, Maruyama S, Ohura T, Hasegawa Y, Toyoshima M, Haapalainen AM, et al. Three Japanese Patients with Beta-Ketothiolase Deficiency Who Share a Mutation, c.431A>C (H144P) in ACAT1 : Subtle Abnormality in Urinary Organic Acid Analysis and Blood Acylcarnitine Analysis Using Tandem Mass Spectrometry. JIMD Rep. 2012;3:107–15. | Original case | 3 | No | N/A | one case described as "growth and development are within normal ranges" | 6.8 | 2.3 | 25 years | No |
| Fukao T, Aoyama Y, Murase K, Hori T, Harijan RK, Wierenga RK, et al. Development of MLPA for human ACAT1 gene and identification of a heterozygous Alu-mediated deletion of exons 3 and 4 in a patient with mitochondrial acetoacetyl-CoA thiolase (T2) deficiency. Mol Genet Metab. 2013;110:184–7. | Original case | 1 | No | N/A | N/A | N/A | 3.4 | 18 years | No |
| Galanello R, Cao A, Olivieri N. Induction of fetal hemoglobin in the presence of increased 3-hydroxybutyric acid associated with beta-ketothiolase deficiency. N Engl J Med. 1994;331:746–7. | Original case | 1 | No | N/A | N/A | N/A | N/A | 3 years | No |
| Gibson KM, Elpeleg ON, Bennett MJ. beta-Ketothiolase (2-methylacetoacetyl-coenzyme A thiolase) deficiency: identification of two patients in Israel. J Inherit Metab Dis. 1996;19:698–9. | Original case | 2 | No | N/A | "normal development" | N/A | N/A | 3 years 6 months | No |
| Gibson KM, Feigenbaum AS. Phenotypically mild presentation in a patient with 2-methylacetoacetyl-coenzyme A (beta-keto)thiolase deficiency. J Inherit Metab Dis. 1997;20:712–3. | Original case | 1 | No | N/A | N/A | N/A | 3.6 | 2 years 11 months | No |
| Gray RG, Lowther GW, Littlewood JM, Middleton B, Bennett MJ. A case of 2-methylacetoacetyl CoA thiolase deficiency with coincidental chromosome abnormalities. J Med Genet. 1984;21:397. | Original case | 1 | No | N/A | N/A | N/A | N/A | 3 years 9 months | No |
| Guo J, Ren D, Guo ZJ, Yu J, Liu F, Zhao RX, et al. Emergence of lesions outside of the basal ganglia and irreversible damage to the basal ganglia with severe beta-ketothiolase deficiency: A case report. World j. 2021;9(30):9276-84. | Original case | 1 | No | N/A | N/A | "normal" | N/A | 1 year 11 months | No |
| Halvorsen S, Stokke O, Jellum E. A variant form of 2-methyl-3-hydroxybutyric and 2-methylacetoacetic aciduria. Acta Paediatr Scand. 1979;68:123–8. | Original case | 1 | No | N/A | "mentally and physically normal" | N/A | N/A | 15 years | No |
| Henry CG, Strauss AW, Keating JP, Hillman RE. Congestive cardiomyopathy associated with beta-ketothiolase deficiency. J Pediatr. 1981;99:754–7. | Original case | 1 | No | N/A | "Height and weight progressed along the twenty-fifth and tenth percentiles, respectively, over the first five years, but subsequently slowed to the third percentile over the next three years." | N/A | N/A | 8 years | No |
| Hillman RE, Keating JP. Beta-ketothiolase deficiency as a cause of the “ketotic hyperglycinemia syndrome.” Pediatrics. 1974;53:221–5. | Urine and fibroblast study using previously published case | 1 | No | N/A | N/A | N/A | N/A | N/A | No |
| Hiyama K, Sakura N, Matsumoto T, Kuhara T. Deficient beta-ketothiolase activity in leukocytes from a patient with 2-methylacetoacetic aciduria. Clin Chim Acta. 1986;155:189–94. | Original case | 1 | No | N/A | N/A | N/A | N/A | 1 year 8 months | No |
| Keating JP, Feigin RD, Tenenbaum SM, Hillman RE. Hyperglycinemia with ketosis due to a defect in isoleucine metabolism: a preliminary report. Pediatrics. 1972;50:890–5. | Original case | 1 | No | N/A | At 10 weeks, weight was 3340 grams. At 1 year, weight was on the 10th centile. | N/A | N/A | 1 year | No |
| Kılıç-Yıldırım G, Durmuş-Aydoğdu S, Ceylaner S, Sass JO. Beta-ketothiolase deficiency: An unusual cause of recurrent ketoacidosis. Turk J Pediatr. 2017;59:471–4. | Original case | 1 | No | N/A | N/A | N/A | 3.6 | 4 years 7 months | No |
| Law C-Y, Lam C-W, Ching C, Yau K-CE, Ho T, Lai C, et al. NMR-based urinalysis for beta-ketothiolase deficiency. Clin Chim Acta. 2015;438:222–5. | Original case | 1 | No | N/A | N/A | N/A | N/A | 1 year | No |
| Leonard JV, Middleton B, Seakins JW. Acetoacetyl CoA thiolase deficiency presenting as ketotic hypoglycemia. Pediatr Res. 1987;21:211–3. | Original case | 2 | No | N/A | Case 1 "thrived and made normal developmental progress until the age of I0 months", Case 2 "well up to the age of 15 months" | N/A | 0.6 | 15 months | No |
| Lin Y, Yang Z, Yang C, Hu H, He H, Niu T, et al. C4OH is a potential newborn screening marker-a multicenter retrospective study of patients with beta-ketothiolase deficiency in China. Orphanet Journal Of Rare Diseases. 2021;16(1):224. | Multi-centre retrospective study | 29 | No | N/A | N/A | N/A | N/A | 9 years 6 months | No |
| Magdy RM, Abd-Elkhalek HS, Bakheet MA, Mohamed MM. Selective screening for inborn errors of metabolism by tandem mass spectrometry at Sohag University Hospital, Egypt. Arch Pediatr. 2022;29(1):36-43. | Cross-sectional study | 1 | No | N/A | N/A | N/A | N/A | N/A | No |
| Manawadu TV, Jasinge E, Fernando M, Gamage P, Gunarathne AV. A Novel Mutation in ACAT1 Causing Beta-Ketothiolase Deficiency in a 4-Year-Old Sri Lankan Boy with Metabolic Ketoacidosis. Indian J. 2020;35(2):251-4. | Original case | 1 | No | N/A | Prior to presentation, "previously well with uneventful birth and neonatal periods and normal development" | N/A | 3.8 | Died ~ 5 years | No |
| Mao S, Yang L, Yin X, Yang J, Huang X. Ketoacidotic crisis after vaccination in a girl with beta-ketothiolase deficiency: a case report. Transl. 2021;10(2):459-63. | Original case | 1 | No | N/A | N/A | 10.7 | N/A | Died < 1 year | No |
| Marquez-Caraveo ME, Ibarra-Gonzalez I, Rodriguez-Valentin R, Ramirez-Garcia MA, Perez-Barron V, Lazcano-Ponce E, et al. Brief Report: Delayed Diagnosis of Treatable Inborn Errors of Metabolism in Children with Autism and Other Neurodevelopmental Disorders. J Autism Dev Disord. 2021;51(6):2124-31. | Substudy of  trial on neuro-  developmental disorders | 1 | No | N/A | N/A | N/A | N/A | 6 years | No |
| Merinero, B, Pérez-Cerdá, C, García, M J, Carrasco, S, Lama, R, Ugarte, M, et al. beta-Ketothiolase Deficiency: Two Siblings with Different Clinical Conditions. J Inherit Metab Dis. 1987;Suppl 2:276–8. | Original case | 2 | No | N/A | "developed normally" | N/A | N/A | 5 years | No |
| Middleton B, Gray RG, Bennett MJ. Two cases of beta-ketothiolase deficiency: a comparison. J Inherit Metab Dis. 1984;7 Suppl 2:131–2. | Original case | 2 | No | N/A | N/A | N/A | N/A | N/A | No |
| Monastiri K, Amri F, Limam K, Kaabachi N, Guediche MN. beta-Ketothiolase (2-methylacetoacetyl-CoA thiolase) deficiency: a frequent disease in Tunisia? J Inherit Metab Dis. 1999;22:932–3. | Original case | 4 | No | N/A | N/A | N/A | N/A | N/A | No |
| Mrázová L, Fukao T, Hálovd K, Gregová E, Kohút V, Pribyl D, et al. Two novel mutations in mitochondrial acetoacetyl-CoA thiolase deficiency. J Inherit Metab Dis. 2005;28:235–6. | Original case | 1 | No | N/A | "development is normal" | N/A | "hypoglycaemia (below the limit of detection)" | 4.5 years | No |
| Nagasawa H, Yamaguchi S, Orii T, Schutgens RB, Sweetman L, Hashimoto T. Heterogeneity of defects in mitochondrial acetoacetyl-CoA thiolase biosynthesis in fibroblasts from four patients with 3-ketothiolase deficiency. Pediatr Res. 1989;26:145–9. | Fibroblast study using previously published cases | 4 | No | N/A | N/A | N/A | N/A | 14 years | No |
| Nakamura K, Fukao T, Perez-Cerda C, Luque C, Song XQ, Naiki Y, et al. A novel single-base substitution (380C>T) that activates a 5-base downstream cryptic splice-acceptor site within exon 5 in almost all transcripts in the human mitochondrial acetoacetyl-CoA thiolase gene. Mol Genet Metab. 2001;72:115–21. | Fibroblast study using previously published cases | 2 | No | N/A | N/A | N/A | N/A | N/A | No |
| Ngu LH, Zabedah MY, Shanti B, Teh SH. Biochemical profiling in two siblings with mitochondrial 2-methylacetoacetyl-CoA thiolase deficiency. Malays J Pathol. 2008;30:109–14. | Original case | 2 | No | N/A | N/A | N/A | "did not develop any hypoglycemia" | 25 months | No |
| O’Neill ML, Kuo F, Saigal G. MRI of pallidal involvement in Beta-ketothiolase deficiency. J Neuroimaging. 2014;24:414–7. | Original case | 1 | No | N/A | N/A | N/A | N/A | 5 years | No |
| Otsuka H, Sasai H, Nakama M, Aoyama Y, Abdelkreem E, Ohnishi H, et al. Exon 10 skipping in ACAT1 caused by a novel c.949G>A mutation located at an exonic splice enhancer site. Mol Med Rep. 2016;14:4906–10. | Original case | 1 | No | N/A | "good health" | N/A | 1.5 | 8 years | No |
| Ozand PT, Rashed M, Gascon GG, al Odaib A, Shums A, Nester M, et al. 3-Ketothiolase deficiency: a review and four new patients with neurologic symptoms. Brain Dev. 1994;16 Suppl:38–45. | Original case and review | 4 | No | N/A | < 5th centile to 10th centile | N/A | N/A | 7 years | No |
| Pang CP, Law LK, Mak YT, Shek CC, Cheung KL, Mak TW, et al. Biochemical investigation of young hospitalized Chinese children: results over a 7-year period. Am J Med Genet. 1997;72:417–21. | Original case | 3 | No | N/A | N/A | N/A | 2 years | N/A | No |
| Paquay S, Bourillon A, Pichard S, Benoist J-F, de Lonlay P, Dobbelaere D, et al. Mitochondrial acetoacetyl-CoA thiolase deficiency: basal ganglia impairment may occur independently of ketoacidosis. J Inherit Metab Dis. 2017;40:415–22. | Retrospective study | 26 | No | N/A | One patient reported as having "weight and growth failure". Another patient "failed to gain weight after the 4th month of life". | N/A | 0.9 | 29 years | No |
| Pollitt RJ. The occurrence of substituted 3-methyl-3-hydroxyglutaric acids in urine in propionic acidaemia and in beta-ketothiolase deficiency. Biomed Mass Spectrom. 1983;10:253–7. | Original case | 1 | No | N/A | N/A | N/A | N/A | 2 years | No |
| Rajan D, Constance LSL, Brandon P. Beta-ketothiolase deficiency in a Malaysian infant. Med J Malaysia. 2019;74:174–5. | Original case | 1 | No | N/A | 3rd centile | "Blood glucose was maintained 4 -12mmol/L". Unclear if glucose infusion was used to maintain BGLs in this range. | N/A | 14 months | No |
| Robinson BH, Sherwood WG, Taylor J, Balfe JW, Mamer OA. Acetoacetyl CoA thiolase deficiency: a cause of severe ketoacidosis in infancy simulating salicylism. J Pediatr. 1979;95:228–33. | Original case | 2 | No | N/A | N/A | N/A | 1.8 | 30 months | No |
| Sabetta G, Bachmann C, Giardini O, Castro M, Gambarara M, Vici CD, et al. beta-Ketothiolase deficiency with favourable evolution. J Inherit Metab Dis. 1987;10:405–6. | Original case | 1 | No | N/A | 10th centile | N/A | N/A | 10 years | No |
| Sakurai S, Fukao T, Haapalainen AM, Zhang G, Yamada K, Lilliu F, et al. Kinetic and expression analyses of seven novel mutations in mitochondrial acetoacetyl-CoA thiolase (T2): identification of a Km mutant and an analysis of the mutational sites in the structure. Mol Genet Metab. 2007;90:370–8. | Original case | 6 | No | N/A | N/A | N/A | N/A | 25 months | No |
| Sarafoglou K, Matern D, Redlinger-Grosse K, Bentler K, Gaviglio A, Harding CO, et al. Siblings with mitochondrial acetoacetyl-CoA thiolase deficiency not identified by newborn screening. Pediatrics. 2011;128:e246-250. | Original case | 3 | No | N/A | N/A | 8.5 | N/A | 4 years | No |
| Sasai H, Aoyama Y, Otsuka H, Abdelkreem E, Nakama M, Hori T, et al. Single-nucleotide substitution T to A in the polypyrimidine stretch at the splice acceptor site of intron 9 causes exon 10 skipping in the ACAT1 gene. Mol Genet Genomic Med. 2017;5:177–84. | Original case and previously published case | 2 | No | N/A | "growth and development normal", "normal development" | N/A | 4.8 | 11 years | No |
| Scolamiero E, Cozzolino C, Albano L, Ansalone A, Caterino M, Corbo G, et al. Targeted metabolomics in the expanded newborn screening for inborn errors of metabolism. Mol Biosyst. 2015;11:1525–35. | Original case in newborn screening study | 1 | No | N/A | N/A | N/A | N/A | N/A | No |
| Shiasi Arani K, Soltani B. First report of 3-oxothiolase deficiency in iran. Int J Endocrinol Metab. 2014;12:e10960. | Original case | 1 | No | N/A | N/A | "normal" | N/A | 20 months | No |
| Søvik O. Mitochondrial 2-methylacetoacetyl-CoA thiolase deficiency: an inborn error of isoleucine and ketone body metabolism. J Inherit Metab Dis. 1993;16:46–54. | Review | 22 | No | N/A | N/A | N/A | N/A | 2 years | No |
| Su L, Li X, Lin R, Sheng H, Feng Z, Liu L. Clinical and molecular analysis of 6 Chinese patients with isoleucine metabolism defects: identification of 3 novel mutations in the HSD17B10 and ACAT1 gene. Metab Brain Dis. 2017;32:2063–71. | Original case | 4 | No | N/A | N/A | N/A | N/A | 13 months | No |
| Sundaram S, Nair M, Namboodhiri S, Menon RN. Mitochondrial acetoacetyl-CoA thiolase enzyme deficiency in a 9-month old boy: Atypical urinary metabolic profile with a novel homozygous mutation in ACAT1 gene. Neurol India. 2018;66:1802–4. | Original case | 1 | No | N/A | N/A | N/A | N/A | 11 months | No |
| Thadchanamoorthy V, Dayasiri K. Unexplained Tachypneoa and Severe Metabolic Acidosis in a Three-Month-Old Child: A Rare Presentation of Beta-Ketothiolose Deficiency. Cureus. 2022;14(2):e21934. | Original case | 1 | No | N/A | 4.8 kg at 3 months, and subsequently "thriving well" | 9.1 | N/A | 3 years 8 months | No |
| Thümmler S, Dupont D, Acquaviva C, Fukao T, de Ricaud D. Different clinical presentation in siblings with mitochondrial acetoacetyl-CoA thiolase deficiency and identification of two novel mutations. Tohoku J Exp Med. 2010;220:27–31. | Original case | 2 | No | N/A | N/A | N/A | N/A | 16 years | No |
| Tilbrook LK, Slater J, Agarwal A, Cyriac J. An unusual cause of interference in a salicylate assay caused by mitochondrial acetoacetyl-CoA thiolase deficiency. Ann Clin Biochem. 2008;45 Pt 5:524–6. | Original case | 1 | No | N/A | N/A | 6.1 | N/A | 18 months | No |
| Vakili R, Hashemian S. A Novel Mutation of Beta-ketothiolase Deficiency: The First Report from Iran and Review of Literature. Iran J Child Neurol. 2018;12:113–21. | Original case | 1 | No | N/A | N/A | "High level of blood sugar" not specified | N/A | 7 months | No |
| Wakazono A, Fukao T, Yamaguchi S, Hori T, Orii T, Lambert M, et al. Molecular, biochemical, and clinical characterization of mitochondrial acetoacetyl-coenzyme A thiolase deficiency in two further patients. Hum Mutat. 1995;5:34–42. | Fibroblast study using previously published cases | 2 | No | N/A | N/A | N/A | N/A | 6 years | No |
| Wang Y, Gao Q, Wang W, Xin X, Yin Y, Zhao C, et al. Identification of two novel ACAT1 variant associated with beta-ketothiolase deficiency in a 9-month-old boy. J Pediatr Endocrinol Metab. 2022;35(9):1194-200. | Original case | 1 | No | N/A | no growth abnormalities | N/A | N/A | 15 months | No |
| Wilcken B, Wiley V, Hammond J, Carpenter K. Screening newborns for inborn errors of metabolism by tandem mass spectrometry. N Engl J Med. 2003;348:2304–12. | Original case in newborn screening study | 1 | No | N/A | N/A | N/A | N/A | 15 months | No |
| Wojcik MH, Wierenga KJ, Rodan LH, Sahai I, Ferdinandusse S, Genetti CA, et al. Beta-Ketothiolase Deficiency Presenting with Metabolic Stroke After a Normal Newborn Screen in Two Individuals. JIMD Rep. 2018;39:45–54. | Original case | 2 | No | N/A | N/A | N/A | N/A | 28 months | No |
| Yalçinkaya C, Apaydin H, Ozekmekçi S, Gibson KM. Delayed-onset dystonia associated with 3-oxothiolase deficiency. Mov Disord. 2001;16:372–5. | Original case | 1 | No | N/A | "uneventful neonatal period and infancy, and achieved normal psychomotor development" | N/A | "hypoglycaemia" not specified | 11 years | No |
| Yamaguchi S, Orii T, Sakura N, Miyazawa S, Hashimoto T. Defect in biosynthesis of mitochondrial acetoacetyl-coenzyme A thiolase in cultured fibroblasts from a boy with 3-ketothiolase deficiency. J Clin Invest. 1988;81:813–7. | Fibroblast study using previously published case | 1 | No | N/A | N/A | N/A | N/A | 20 months | No |
| Yang Y, Jiang SH, Liu S, Han XY, Wang Y, Wang LL, et al. Two Infants With Beta-Ketothiolase Deficiency Identified by Newborn Screening in China. Front Genet. 2019;10:451. | Original case | 2 | No | N/A | birth weight 3800 grams | N/A | 2.1 | 15 months | No |
| Zhang GX, Fukao T, Rolland M-O, Zabot M-T, Renom G, Touma E, et al. Mitochondrial acetoacetyl-CoA thiolase (T2) deficiency: T2-deficient patients with “mild” mutation(s) were previously misinterpreted as normal by the coupled assay with tiglyl-CoA. Pediatr Res. 2004;56:60–4. | Original cases and previously published cases | 4 | No | N/A | N/A | N/A | N/A | 19 months | No |
| Zhang G, Fukao T, Sakurai S, Yamada K, Michael Gibson K, Kondo N. Identification of Alu-mediated, large deletion-spanning exons 2-4 in a patient with mitochondrial acetoacetyl-CoA thiolase deficiency. Mol Genet Metab. 2006;89:222–6. | Original case | 1 | No | N/A | N/A | N/A | N/A | 14 months | No |

*Please note articles were sorted alphabetically by the first author's surname, then by year of publication if the first authors' surnames were identical, then alphabetically by the second author's surname.
